# Production, Purification, and Characterization of a Major *Penicillium glabrum* Xylanase Using Brewer's Spent Grain as Substrate

**DOI:** 10.1155/2013/728735

**Published:** 2013-05-13

**Authors:** Adriana Knob, Susan Michelz Beitel, Diana Fortkamp, César Rafael Fanchini Terrasan, Alex Fernando de Almeida

**Affiliations:** ^1^Department of Biological Sciences, Midwest State University, Camargo Varela de Sá Street 03, 85040-080 Guarapuava, PR, Brazil; ^2^Department of Biology, Midwest State University, Camargo Varela de Sá Street 03, 85040-080 Guarapuava, PR, Brazil; ^3^Department of Chemical Engineering, Federal University of São Carlos, Rodovia Washington Luís, km 235, SP-310, 13565-905 São Carlos, SP, Brazil; ^4^Department of Biochemistry and Microbiology, São Paulo State University, 24-A Avenue 1515, 13506-900 Rio Claro, SP, Brazil

## Abstract

In recent decades, xylanases have been used in many processing industries. This study describes the xylanase production by *Penicillium glabrum* using brewer's spent grain as substrate. Additionally, this is the first work that reports the purification and characterization of a xylanase using this agroindustrial waste. Optimal production was obtained when *P. glabrum* was grown in liquid medium in pH 5.5, at 25 °C, under stationary condition for six days. The xylanase from *P. glabrum* was purified to homogeneity by a rapid and inexpensive procedure, using ammonium sulfate fractionation and molecular exclusion chromatography. SDS-PAGE analysis revealed one band with estimated molecular mass of 18.36 kDa. The optimum activity was observed at 60 °C, in pH 3.0. The enzyme was very stable at 50 °C, and high pH stability was verified from pH 2.5 to 5.0. The ion Mn^2+^ and the reducing agents **β**-mercaptoethanol and DTT enhanced xylanase activity, while the ions Hg^2+^, Zn^2+^, and Cu^2+^ as well as the detergent SDS were strong inhibitors of the enzyme. The use of brewer's spent grain as substrate for xylanase production cannot only add value and decrease the amount of this waste but also reduce the xylanase production cost.

## 1. Introduction

Xylan is a major structural polysaccharide of plant-cell walls being the second most prevalent in nature after cellulose. It is a heterogeneous polymer constituted primarily by a linear *β*-(1,4)-D-xylose backbone, which is partially acetylated and substituted at different degrees by a variety of side chains, mainly *α*-D-glucuronosyl and *α*-L-arabinosyl units. Due to its structural complexity, several hydrolases are required for complete xylan degradation. The key enzyme in this process is endo-*β*-(1,4)-xylanase (EC 3.2.1.8), which cleaves the xylan backbone to xylooligosaccharides [1, 2]. 

Interest in xylanolytic enzymes has increased in recent years due to their potential application in biotechnology. Xylanolytic enzymes have applications in conversion of lignocellulosic materials to chemicals and fuels, animal feed digestion, food and textile industries, and as bleaching agents in the pulp and paper processing [3, 4].

Industrial production of enzymes on large scale is associated mainly with the substrate. The use of agroindustrial wastes as low-cost substrates for the industrial enzymes production is a significant way to reduce production cost. Thus, lignocellulosic substrates have now received considerable interest because of their possible use in secondary fermentation process [5]. Various lignocellulosic substrates involving agroindustrial waste materials like corn meal, corn cob, wheat bran, wheat straw, rice straw, sugarcane bagasse, and coffee by-products are being used as substrates for the production of fungal xylanases [6–8]. 

Brewer's spent grain (BSG), the main residue of brewing industry, is rich in cellulosic and noncellulosic polysaccharides. It is produced at large amounts during the year and represents around 85% of the total by-products generated [9]. In Brazil, the world's fourth largest beer producer (8.5 billion litres/year), approximately 1.7 million tonnes of BSG were generated per year. Despite the large amounts produced, BSG has received little attention as a low-cost by-product and its use is still limited, mainly as animal feed [10]. 

*Penicillium glabrum* is a filamentous fungus that is distributed worldwide, frequently involved in food contamination. Due to its ability to disperse a large number of spores in the environment, *P. glabrum* is frequently found in the food manufacturing industry [11]. Despite its large implication in food contamination, few studies have been conducted to investigate the enzyme production by this fungus. Only tannase production by *P. glabrum* has been described in the literature [12]. Since many agroindustrial wastes are a potentially valuable resource for industrial exploitation, this work aimed to evaluate the xylanase production by *P. glabrum* using different agroindustrial wastes, establish the best fungal growing conditions for xylanase production with the best substrate, and biochemically characterize the major enzyme purified.

## 2. Materials and Methods

### 2.1. Organism and Growth

*P. glabrum* used in the present work is available in the Culture Collection of Environmental Studies Center—CEA/UNESP, SP, Brazil. Conidia were obtained from cultures in Vogel solid medium [13] containing 1.5% (w/v) glucose and 1.5% (m/v) agar at 25 °C for 7 days. Liquid cultures were prepared in the same medium with 1% (w/v) carbon source and the pH was adjusted to 6.5. Erlenmeyer flasks (125 mL) containing 25 mL of medium were inoculated with 1.0 mL of spore suspension containing 5 × 10^7^ spores/mL and incubated at 30 °C for 5 days in stationary condition. 

### 2.2. Preparation of Agroindustrial Wastes

The agroindustrial wastes were obtained locally. The residues were prepared by exhaustive washing with distilled water, dried at 80 °C for 24–48 h, and milled (35 mesh).

### 2.3. Enzyme Preparations and Assays

Cultures were harvested by filtration. The culture filtrate was assayed for extracellular activity and protein. The mycelium was washed with distilled and sterilized water, frozen, and ground with sand in 0.05 M sodium phosphate buffer pH 6.5. The slurry was centrifuged at 3.900 g at 4 °C, and the supernatant was used as intracellular protein source.

Xylanase activity was determined at 50 °C using 1.0% (w/v) birchwood xylan (Sigma, st. Louis, MO, USA) in McIlvaine buffer pH 6.5. This buffer is prepared by a mixture of 0.1 M citric acid and 0.2 M sodium monohydrogen phosphate. After 5 and 10 min of incubation, the reaction was interrupted by the addition of 3,5-dinitrosalicylic acid (DNS), and the reducing sugars released were quantified [14], using xylose as standard. One unit of enzyme activity was defined as the amount of enzyme capable of releasing 1 *μ*mol of reducing sugar per min, under assay conditions. Specific activity was expressed as unit per milligram of protein. All enzymatic assays were developed in triplicate, and the results are presented through mean values. 

### 2.4. Protein Determination

Total protein was determined by modified Bradford method [15], using bovine serum albumin (BSA) as standard. 

### 2.5. Culture Conditions for Xylanase Production

#### 2.5.1. Enzyme Production on Different Carbon Sources

Vogel's liquid medium was supplemented with various carbon sources at a concentration of 1% (w/v). The inoculated flasks were incubated at 28 °C under stationary condition, for five days. Xylanase activity was determined as described previously. 

#### 2.5.2. Effect of Incubation Period, Initial pH, and Temperature on Xylanase Production

The incubation period's influence on xylanase production by *P. glabrum* was studied under standing culture and under shaking culture (120 rpm) for 8 days. The effect of initial pH on the enzyme production was assayed from 3.0 to 8.0, and the temperature influence was verified from 15 to 40 °C. The initial pH values were adjusted by the addition of 1.0 M sodium hydroxide or phosphoric acid solutions. 

### 2.6. Purification of a Major *P. glabrum* Xylanase

#### 2.6.1. Ammonium Sulfate Fractionation

The crude enzyme (50 mL) was fractionated by ammonium sulfate precipitation (0–90%, w/v). The supernatant of 90% ammonium sulfate saturation obtained after centrifugation (6.000 g, 20 min., 4 °C) was extensively dialyzed against 0.05 M ammonium acetate buffer pH 6.8 before analyses. 

#### 2.6.2. Exclusion Molecular Chromatography

The protein sample obtained in the step above was chromatographed on Sephadex G-75 column (2.6 × 64.0 cm), equilibrated, and eluted with 0.05 M ammonium acetate buffer, pH 6.8, flowing at 18 mL/h. Fractions (3 mL) whose protein content was estimated by reading absorbance at 280 nm and xylanase activity assayed as described previously. To determine xylanase molecular mass through gel filtration chromatography, the column was calibrated using blue dextran for the void volume determination and ribonuclease (15.4 kDa), chymotrypsin (25.0 kDa), ovalbumin (43.0 kDa) and bovine serum albumin (67.0 kDa) as standards. The molecular weight of xylanase was estimated from a regression curve (*R* ^2^ = 0.993), by plotting log of the molecular weights of the standards against the ratio between elution volumes of the standards and the void volume of the column.

#### 2.6.3. Electrophoresis

Sodium dodecyl sulfate-polyacrylamide gel electrophoresis (SDS-PAGE) was performed using a gradient of 8–18% (w/v) polyacrylamide according to Laemmli [16]. The resolved protein bands were visualized after staining with 0.1% (w/v) Coomassie Brilliant Blue R-250 in methanol, acetic acid, and distillated water (4 : 1 : 5, v/v/v). The proteins phosphorylase b, bovine serum albumin, ovalbumin, carbonic anhydrase, trypsin inhibitor, and *α*-lactalbumin (SDS-LMW markers-Sigma, st. Louis, MO, USA) were used to plot the standard curve log of molecular weight against relative mobility in the gel. 

### 2.7. Enzyme Characterization

#### 2.7.1. Temperature and pH Optima, Thermal and pH Stability

To determine the optimum pH, the purified xylanases were assayed at 50 °C, in different pH values using 0.05 M glycine-HCl buffer from pH 1.6 to 2.5 and McIlvaine buffer from 3.0 to 7.0. The optimum temperatures were determined by performing the reaction at temperatures ranging from 15 °C to 75 °C in McIlvaine buffer pH 3.0. 

For pH stability assays, the purified enzymes were diluted (1 : 2 v/v) in 0.05 M glycine-HCl buffer from pH 1.6 to 2.5 and McIlvaine buffer for pH range from 3.0 to 7.0. The samples were incubated at 4 °C for 24 h. After this period, the xylanase activity was assayed under optimal conditions. To evaluate the thermal stability, the purified enzyme was incubated at 50 °C, 55 °C, and 60 °C at the optimal pH determined above for different periods. 

#### 2.7.2. Effect of Substances

The effect of metallic ions and other compounds on the xylanase activity was evaluated at concentrations of 2 mM and 10 mM. The residual activities were measured in relation to the control without substances by performing the enzyme assay at the optimal conditions. 

#### 2.7.3. Substrate Specificity

Specificity of xylanase against birchwood, beechwood, and oat spelt xylans, carboxymethyl cellulose, (CM-cellulose) and Avicel were assayed. Substrate solutions of 1% (w/v) were prepared in a buffer of optimum pH activity for the enzyme. 

#### 2.7.4. Kinetic Parameters

The enzyme was incubated with oat spelt, beechwood, and birchwood xylans, at concentrations between 4.0 and 30 mg/mL. The Michaelis-Menten constant (*K*_*m*_) and maximum reaction velocity (*V*_max⁡_) were estimated from the Lineweaver-Burk reciprocal plots, using “GraFit” 5.0 software. 

## 3. Results and Discussion 

### 3.1. Influence of the Carbon Source on Xylanase Production

Distinct substrates, such as pure carbohydrates and some agroindustrial wastes, were evaluated for xylanase production (Table 1). Among the pure carbohydrates, highest values of xylanase activity were obtained with oat spelt xylan, corresponding to 25.44 U/mL and 64.96 U/mg protein. In general, higher levels of xylanolytic enzymes can be achieved with substrates derived from xylan. According to Kulkarni et al. [1], xylanase activity is inducible and xylan-rich substrates play an important role in xylanase induction. When *P. glabrum* was cultivated in medium with lactose, sucrose, cellobiose, Avicel, and CM-cellulose, enzyme activity was not detected. Conversely, lactose and sucrose increased xylanase production by *Penicillium canescens* 10-10c [17]. In the presence of glucose, xylose, and maltose only low levels of enzyme activity were verified, when compared to the cultures with oat spelt xylan.

Among the agricultural and agroindustrial wastes, highest xylanase production was observed with brewer's spent grain (34.32 U/mL and 102.65 U/mg protein). These values were higher than those obtained with oat spelt xylan. The production of xylanolytic enzymes by *Penicillium janczewskii* with brewer's spent grain as substrate was previously described by Terrasan et al. [18]. However, lower levels of xylanase activity were obtained when compared to this study, corresponding to 15.8 U/mL, under optimized conditions. Xylanase activity was also observed in the presence of wheat bran, oat bran, and rice straw; however, the values obtained, were lower than that observed by brewer's spent grain. Only low levels of xylanase activity were verified with sugarcane bagasse, soybean meal; and corn cobs. Orange bagasse and citrus pectin provided minimal fungal growth, with absent or no significant levels of xylanase produced. The different production levels observed among the lignocellulosic materials are probably related on differences in composition and the accessibility of the substrates to the fungi. Considering the elevated xylanase production obtained with BSG, this substrate was selected for the subsequent optimization experiments.

### 3.2. Effects of Culture Conditions on Xylanase Production

**Figure 1:**
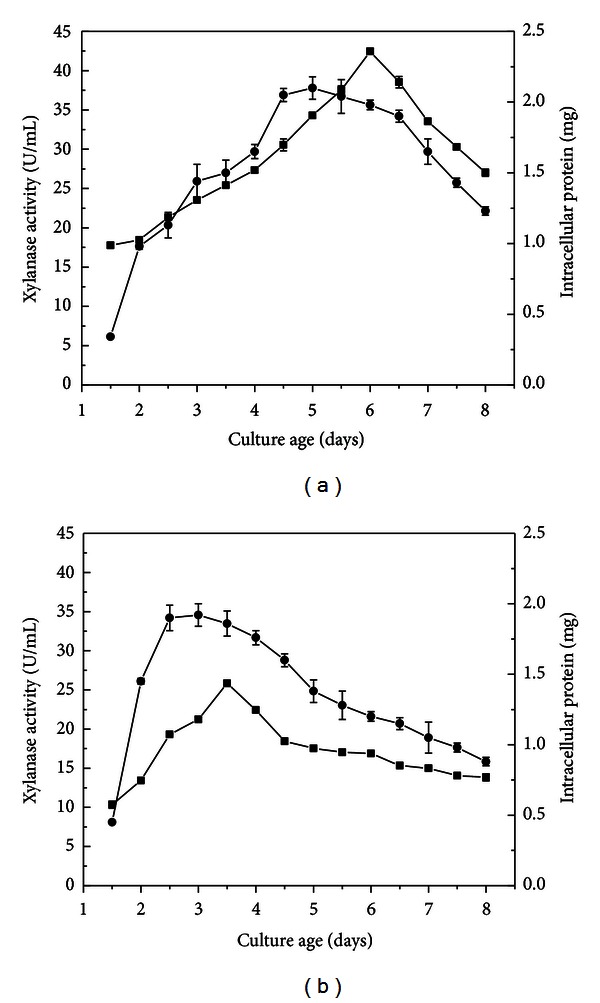
Time course of xylanase production by *P. glabrum* in stationary (a) and shake culture at 120 rev min^−1^ (b). Culture conditions: Vogel medium with 1% (w/v) brewer's spent grain, pH 6.5 at 28 °C. (■) Xylanase activity (U/mL), (•) intracellular protein (mg).

Cultivation conditions are essential for the successful production of an enzyme, and optimization of parameters such as period of cultivation, pH, and temperature is important for a process development. In standing culture (Figure 1(a)) with brewer's spent grain, the highest xylanase production was verified in 6.0 day-old cultures (42.45 U/mL). In shaking condition (Figure 1(b)), maximal xylanase activity was observed on 3.5-day-old cultures, corresponding to the values of 25.85 U/mL. The highest *P. glabrum* growth, measured by the intracellular protein concentration, occurred on the fifth day in standing culture and on the third day in shake culture. Under shaking, as well as under standing condition, xylanases were expressed during the stationary phase, reaching the decline phase (Figure 1). 

**Figure 2:**
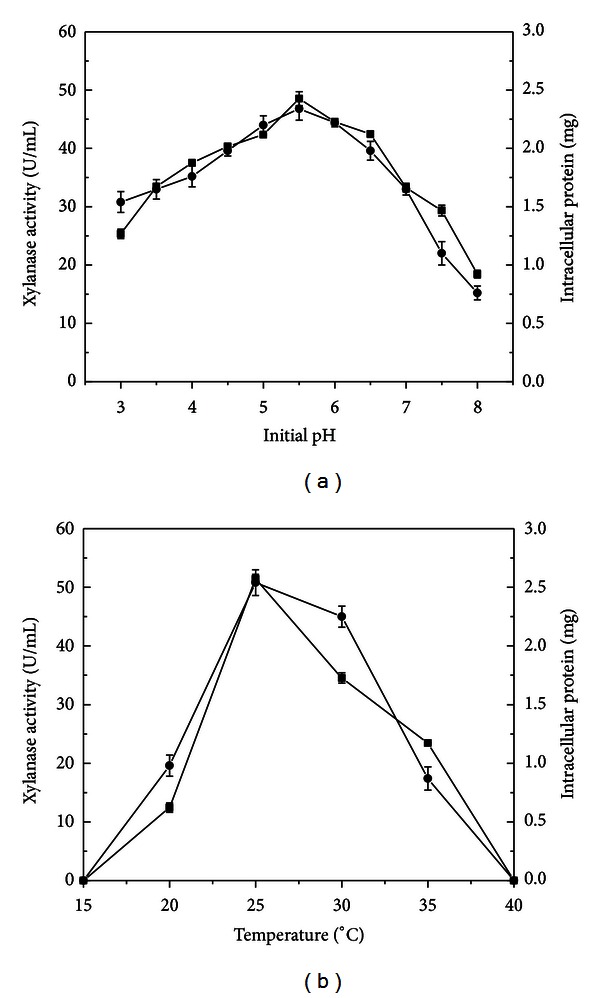
Effect of initial pH (a) and temperature (b) on xylanase production by *P. glabrum.* Culture conditions: Vogel medium with 1% (w/v) brewer's spent grain under stationary condition for six days at 28 °C (a) and pH 5.5 (b). (■) Xylanase activity (U/mL), (•) intracellular protein (mg).

Temperature and pH are important environmental parameters that determine growth rates of microorganisms and significantly affect the level of xylanases produced. The influence of pH culture on xylanase production during *P*. *glabrum* cultivation is presented in Figure 2(a). Xylanase activity was detected in all pH evaluated. The highest activity was observed at initial pH 5.5, corresponding to the values of 48.54 U/mL. With rare exceptions, xylanase production by filamentous fungi occurs in cultures with an initial pH under 7.0. *Trichoderma harzianum* [19] and *Penicillium janczewskii* [18] showed enhanced xylanase production at pH 5.0 and 5.5, respectively. *P. glabrum* could grow in media with initial pH between 3.0 and 8.0 (Figure 2(a)), with maximal growth in the range of 4.5 to 6.5. This result clearly indicates the acidophilic nature of this fungus.

The effect of temperature on xylanase production by *P. glabrum* is presented in Figure 2(b). The highest xylanase activity was verified at 25 °C, corresponding to 51.43 U/mL. Similarly, maximum xylanase production by *Trichoderma viride* was achieved at 25 °C [20]. The optimal growth verified at 25 °C is in accordance with the literature that describes this temperature as ideal for *P. glabrum* [11]. The maximal temperature for this filamentous fungus was 35 °C, which is in agreement with some data reporting the absence of growth above 37 °C [21, 22]. 

### 3.3. Purification of Xylanase

**Figure 3:**
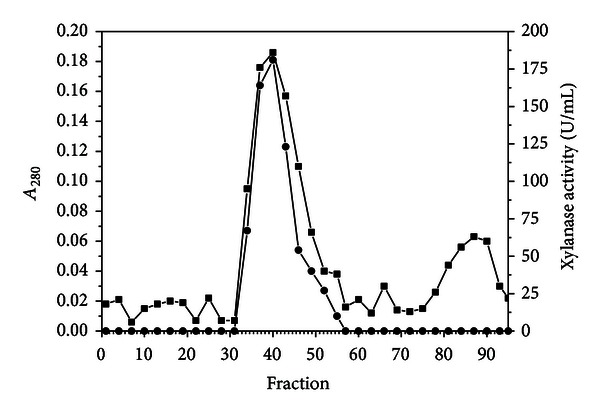
Gel filtration on Sephadex G-75 of the xylanase from *P. glabrum*. The column was equilibrated and eluted with 50 mM ammonium acetate buffer pH 6.8. The flow rate and fraction size were 18 mL/h and 3.0 mL, respectively. (■) *A*_280_ and (•) xylanase activity.

**Figure 4:**
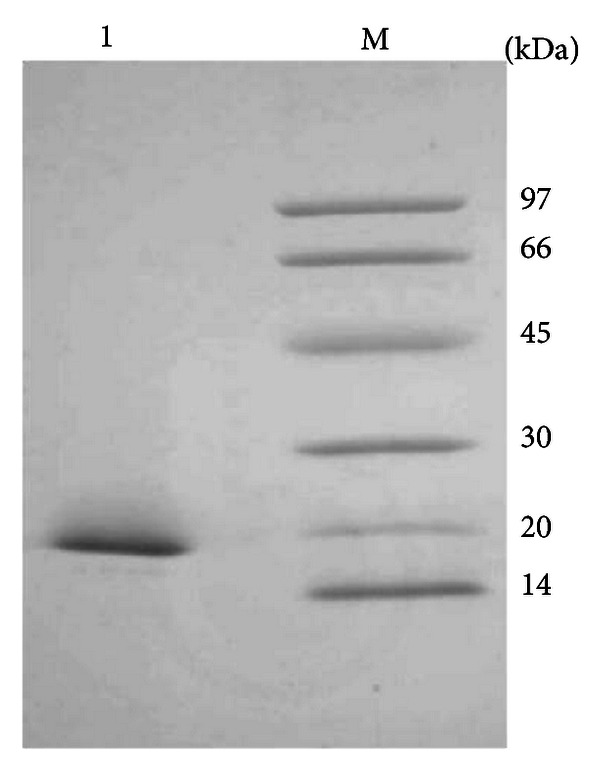
SDS-PAGE (8–18%) of the purified *P. glabrum *xylanase. Lane M: low molecular weight standard proteins; lane 1: purified enzyme.

The major *P. glabrum* xylanase was purified by protein precipitation with ammonium sulfate and molecular exclusion chromatography. High enzyme proportion (about 84%) was observed in the 90% ammonium sulfate saturation supernatant. The molecular exclusion chromatography elution profile resulted in one xylanase activity peak (Figure 3). The fractions corresponding to this peak were pooled and the sample was submitted to SDS-PAGE, showing electrophoretic homogeneity (Figure 4).

The electrophoretic analysis revealed that xylanase corresponded to a single molecular mass band of 18.36 kDa. Native enzyme molecular mass of 21.3 kDa was estimated for *P. glabrum* xylanase by molecular exclusion chromatography, showing monomeric form. The molecular mass of the* P. glabrum* xylanase is in agreement with those found for the catalytic domain of low molecular mass xylanases, belonging to family 11 [23]. Sanghvi et al. [7] partially purified a xylanase from *T. harzianum* with 29.0 kDa molecular mass, while 27.0 kDa was the molecular mass estimated for the xylanase from *Penicillium* sp. [8].

The summary of the *P. glabrum *xylanase purification is presented in Table 2. The procedure resulted in an overall yield of 76.94%, and the specific activity increased 5.10-fold. The purified xylanase exhibited high specific activity corresponding to 457.89 U mg^−1^ protein. 

### 3.4. Properties of Purified Xylanase

#### 3.4.1. Effects of pH and Temperature, Thermal and pH Stabilities

**Figure 5:**
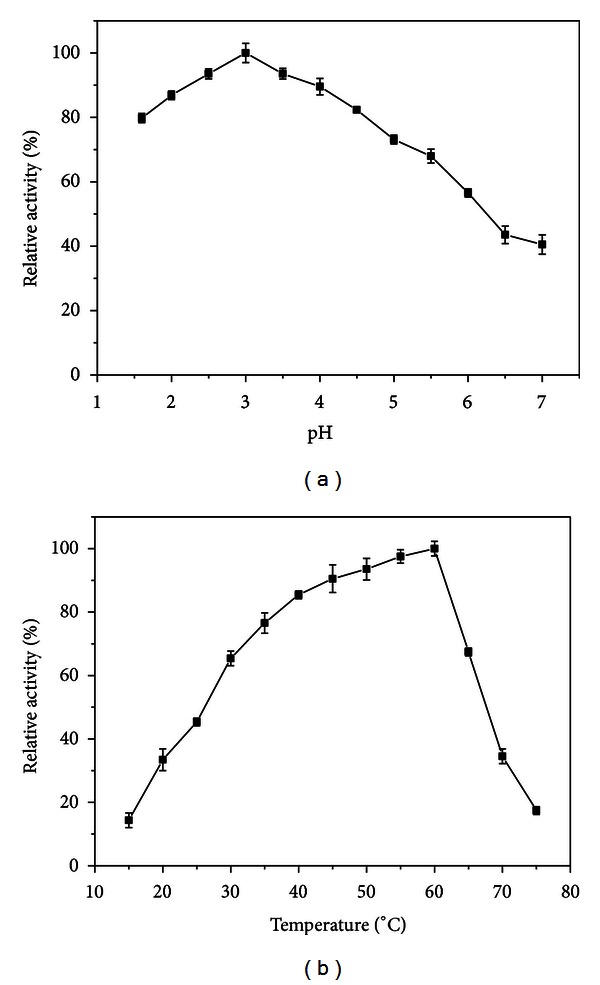
Influence of pH (a) and temperature (b) on the purified *P. glabrum *xylanase. Assay conditions: 0.05 M glycine-HCl buffer from pH 1.6 to 2.5 and McIlvaine buffer from 3.0 to 7.0; 50 °C (a); McIlvaine buffer pH 3.0 (b).

The effects of temperature and pH on the activity of the purified xylanase were investigated. The xylanase showed optimal activity at pH 3.0 (Figure 5(a)). Similarly, *Laetiporus sulphureus* xylanase exhibited optimum activity at pH 3.0 [24]. However, most xylanases present optimal activity in pH between 5.0 and 7.0 [25], and among the acidophilic xylanases, the majority of them showed high activity only under slight acid conditions. 

The optimum temperature for xylanase activity was 60 °C (Figure 5(b)). Values of optimum temperature of xylanase hydrolysis vary according to the producing microorganism. Usually, xylanases from filamentous fungi show optimum temperature between 40 °C and 55 °C [26–28]. Nevertheless, other fungal xylanases show optimum temperature at 60 °C or above [24, 29]. 

**Figure 6:**
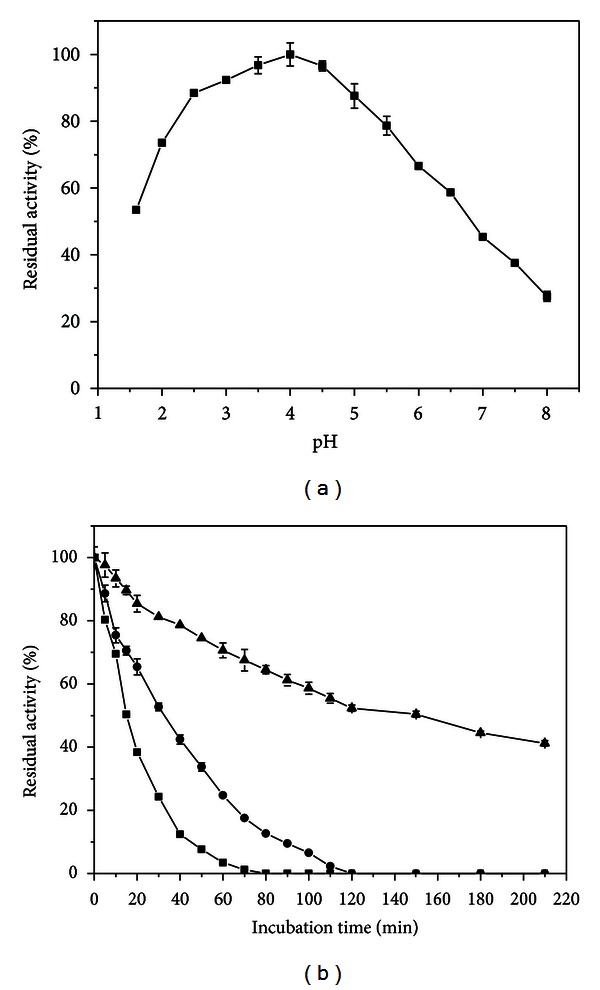
pH (a) and thermal (b) stabilities of purified *P. glabrum* xylanase. The enzymatic preparation was incubated without substrate with glycine-HCl buffer from pH 1.6 to 2.5 and McIlvaine buffer from pH 3.0 to 7.0 at 4 °C for 24 h (a); the enzymatic preparation was incubated at (▲) 50, (•) 55, and (■) 60 °C, without substrate (b). In both assays, the residual xylanase activity was assayed with McIlvaine buffer, pH 3.0, at 60 °C.

A pH stability study is an essential part of an enzyme characterization before it can be exploited commercially. The xylanase produced by *P. glabrum* was stable over a broad pH range (Figure 6(a)). Xylanase activity was maintained over 80% at pH from 2.5 to 5.0. Microbial xylanases are usually stable over a wide pH range (3–10) [1]. The optimum activity in very acidic conditions and pH stability exhibited by *P. glabrum* xylanase make this enzyme attractive for some industrial applications, such as in feed and food industries.

Thermal stability is an interesting enzyme property due to the great industrial importance. Then, enzyme stability analyses were carried out. The purified xylanase from *P. glabrum* was incubated without substrate at 50 °C, 55 °C, and 60 °C (Figure 6(b)). The estimated half-lives (*T*_1/2_) at 60 °C and 55 °C were 15 and 32 min, respectively. This enzyme was stable at 50 °C with *T*_1/2_ of 150 min, retaining 70% of its activity over 60 min at this temperature. The *P. glabrum* xylanase is more thermostable than many fungal xylanases, such as those from *Penicillium expansum* [27] and *Aspergillus niger* B03 [28], however, less thermostable than the xylanase from thermophilic *Talaromyces thermophilus* [30]. 

#### 3.4.2. Effect of Substances

In order to verify the effect of substances on xylanase activity, the purified enzyme was incubated in the presence of several metallic ions, sodium dodecyl sulfate (SDS), tetrasodium ethylenediaminetetraacetate (EDTA), dithiothreitol (DTT), phenylmethylsulfonyl fluoride (PMSF), and *β*-mercaptoethanol, at 2 mM and 10 mM concentrations (Table 3). In general, the xylanase activity was enhanced with increased concentration of the substances used. Hg^2+^, Cu^2+^, and Zn^2+^ were strong inhibitors of the xylanase. Likewise, *Penicillium sclerotiorum* and *Aspergillus ficuum* xylanases were inhibited by these ions [26, 31]. The inhibition by Hg^2+^ seems to be a general property of xylanases, indicating the presence of thiol groups of cysteine residues in their active sites or around them [32]. Xylanase activity remains unaltered in the presence of Na^+^ and Mg^2+^. Slight activation was observed in the presence of Ca^2+^, Co^2+^, and Ba^2+^. Additionally, *P. glabrum* xylanase was remarkably stimulated when incubated with Mn^2+^, as *A. niger* B03 xylanase [28].

EDTA, a metal chelator, decreased xylanase activity, indicating that the purified enzyme requires metal ions for their actions. Total loss of activity was observed in the presence of SDS, indicating that hydrophobic interactions must be important in maintaining xylanase structure. The reducing agents *β*-mercaptoethanol and DTT stimulated xylanase activity. The enzymatic activity stimulation in the presence of these thiol group-protecting agents can be explained by preventing the oxidation of sulfhydryl groups. Dutta et al. [33] and Cardoso and Filho [34] also related the involvement of cysteine residues in the maintenance of tertiary structure of the active site in *Penicillium citrinum* and *Acrophialophora nainiana* xylanases, respectively.

#### 3.4.3. Substrate Specificity and Kinetic Studies

Specificity studies indicated that the *P. glabrum* xylanase did not hydrolyze Avicel or CM-cellulose but acted only on xylans. The purified xylanase exhibited typical Michaelis-Menten kinetics for oat spelt, birchwood, and beechwood xylans, allowing the corresponding kinetic constants to be calculated. *P. glabrum* xylanase showed *K*_*m*_ values of 5.3, 3.1, and 1.2 mg mL^−1^ and *V*_max⁡_ values of 212.10, 194.21, and 393.17 *μ*mol min^−1^ mg^−1^ of protein, for birchwood, beechwood, and oat spelt xylans, respectively. The *K*_*m*_ and *V*_max⁡_ values exhibited by *P. glabrum* xylanase are in agreement with the values presented by other fungal xylanases which range from 0.09 to 40.9 mg mL^−1^ for *K*_*m*_ and from 0.106 to 10.000 for *V*_max⁡_ [4]. The values of *K*_*m*_ for these substrates indicated that this enzyme has higher affinity for oat spelt xylan. Similarly, the xylanases from *A. nainiana* [34] and *Fusarium oxysporum* [35] showed highest value of *K*_*m*_ for oat spelt xylan. 

## 4. Conclusions

In this study, a *P. glabrum* strain was able to produce high levels of xylanase using brewer's spent grain as substrate. Conventional purification methods were effective to purify the major xylanase from *P. glabrum*. The purification procedure resulted in higher overall yields as compared to others described in the literature [24, 26, 36]. Furthermore, the specific activity of purified xylanase is higher than those of previously purified xylanases [26, 31, 37]. *P. glabrum* xylanase was active at very low pH, with optimum at 3.0, and it was stable in an acid pH range. These characteristics make it potentially useful in some biotechnological processes such as for animal feed, clarification, and maceration of juices and wines. Additionally, the use of brewer's spent grain as substrate for xylanase production can not only add value and decrease the amount of this waste but also reduce xylanase production cost.

## Figures and Tables

**Table 1:** Influence of pure carbohydrates and agroindustrial wastes on xylanase production by *P. glabrum*.

Carbon source (1% w/v)	Intracellular protein (mg)	Xylanase activity	
		(U/mL)	(U/mg protein)
Pure carbohydrates			

Glucose	1.75	0.04	0.07
Xylose	1.04	0.25	0.60
Maltose	0.89	0.06	0.14
Lactose	0.34	ND	ND
Sucrose	1.34	ND	ND
Cellobiose	1.11	ND	ND
Avicel	0.25	ND	ND
CM-cellulose	0.34	ND	ND
Oat spelt xylan	1.98	25.44	64.96

Agroindustrial wastes			

Sugarcane bagasse	0.53	8.34	25.14
Wheat bran	1.84	20.54	43.35
Oat bran	1.14	13.54	28.31
Rice straw	1.24	17.65	30.34
Brewer's spent grain	2.10	34.32	102.65
Soybean meal	0.76	8.23	20.43
Corn cobs	0.83	7.32	18.23
Citrus pectin	0.34	0.10	0.34
Orange bagasse	0.38	0.16	0.65

**Table 2:** Purification of xylanase from *P. glabrum. *

Purification step	Total activity (U)	Total protein (mg)	Specific activity (U/mg protein)	Purification (fold)	Recovery (%)
Culture filtrate	2571.50	28.61	89.84	1.00	100.00
Supernatant 90% of (NH_4_)_2_SO_4_	2165.01	7.42	291.78	3.25	84.19
Sephadex G-75	1978.10	4.32	457.89	5.10	76.92

**Table 3:** Effect of different substances on relative activity (%) of purified xylanase from *P. glabrum*.

	Relative activity (%)	
Substance	Concentration	
	2 mM	10 mM
Control	100	100
CuCl_2_	69.7 ± 0.3	21.3 ± 0.3
ZnSO_4_	84.4 ± 2.0	75.2 ± 1.1
MnSO_4_	178.9 ± 1.6	206.7 ± 2.2
BaCl_2_	117.5 ± 1.5	124.9 ± 1.7
CaCl_2_	122.0 ± 1.4	126.5 ± 1.2
NH_4_Cl	102.0 ± 1.55	134.3 ± 1.7
NaCl	99.3 ± 1.3	99.2 ± 1.5
SDS	ND	ND
PMSF	92.0 ± 0.9	89.3 ± 0.5
MgSO_4_	97.2 ± 1.7	97.0 ± 1.2
DTT	113.4 ± 1.2	134.4 ± 2.0
CoCl_2_	117.2 ± 1.6	122.2 ± 1.4
HgCl_2_	47.1 ± 0.5	ND
Pb(CH_3_COO)_2_	68.7 ± 2.0	19.1 ± 0.6
EDTA	89.0 ± 2.0	64.8 ± 1.0
*β*-mercaptoethanol	124.0 ± 2.0	136.0 ± 2.0
